# Comprehensive expression profiles of gastric cancer molecular subtypes by immunohistochemistry: implications for individualized therapy

**DOI:** 10.18632/oncotarget.10115

**Published:** 2016-06-16

**Authors:** Hyo Song Kim, Su-Jin Shin, Seung-Hoon Beom, Minkyu Jung, Yoon Young Choi, Taeil Son, Hyoung-Il Kim, Jae-Ho Cheong, Woo Jin Hyung, Sung Hoon Noh, Hyunsoo Chung, Jun Chul Park, Sung Kwan Shin, Sang Kil Lee, Yong Chan Lee, Woong Sub Koom, Joon Seok Lim, Hyun Cheol Chung, Sun Young Rha, Hyunki Kim

**Affiliations:** ^1^ Division of Medical Oncology, Department of Internal Medicine, Yonsei University College of Medicine, Seoul, Republic of Korea; ^2^ Department of Pathology, Yonsei University College of Medicine, Seoul, Republic of Korea; ^3^ Department of Surgery, Yonsei University College of Medicine, Seoul, Republic of Korea; ^4^ Division of Gastroenterology, Department of Internal Medicine, Yonsei University College of Medicine, Seoul, Republic of Korea; ^5^ Department of Radiation Oncology, Yonsei University College of Medicine, Seoul, Republic of Korea; ^6^ Department of Radiology, Yonsei University College of Medicine, Seoul, Republic of Korea

**Keywords:** gastric cancer, molecular subtypes, immunohistochemistry, in-situ hybridization

## Abstract

Gastric cancer (GC) is a leading cause of death. We aim to establish a clinically relevant assay that encompasses recent molecular classifications and provides useful clinical information in a large cohort of GC patients. A consecutive series of 438 GC patients that underwent palliative chemotherapy between 2014 and 2015 were assessed using 10 GC panels: EBER in-situ hybridization, immunohistochemistry for mismatch repair (MMR) proteins (MLH1, PMS2, MSH2, and MSH6), receptor tyrosine kinases (RTKs; HER2, EGFR, and MET), PTEN, and p53 protein. With a median of one aberration, 3.3 % of samples analyzed were Epstein-Barr virus (EBV)-positive; 4.8%, MMR-deficient. RTKs were overexpressed in 218 patients; EGFR was most commonly overexpressed (39.9%), followed by HER2 (13.5%) and MET (12.1%). Furthermore, 2.5 % and 10.7 % of cases had simultaneous overexpression of three and two RTKs, respectively. p53 overexpression/null tumors were identified in 259 patients (59.1%), and PTEN loss was identified in 89 patients (20.3%). EBV-positivity was mutually exclusive with MMR-deficiency, predominantly identified in male patients, and these tumors were undifferentiated with proximal location. p53 mutant type was significantly found predominantly in the EBV-negative (60.6% vs 14.3%, P=0.001) and HER2-positive (78.0% vs 56.2%, P=0.002) groups. We described a molecular spectrum of distinct GC subtypes using clinically applicable assay. This assay will provide a convenient screening tool and facilitate the development of targeted agents in clinical trials.

## INTRODUCTION

Gastric cancer (GC) is the second-leading cause of cancer-related deaths, and more than half of those cases occur in East Asia [1]. Despite current treatment efforts involving surgical resection combined with adjuvant chemotherapy, 25-40% of stage II-IV patients experience relapse [2–4]. Cancer research over the past decade has neglected to focus on the heterogeneity of GC, and patients at more advanced stages are typically treated with 5-fluorouracil/cisplatin-based chemotherapy. Traditional efforts for proper classification of GC with anatomic sites [5] and histopathology, such as Lauren or World Health Organization classification, have little therapeutic relevance. Therefore, active molecular classification has been recently introduced to develop more specific treatments for GC.

In the ToGA trial [6], HER2-positive GC patients who received first-line treatment with trastuzumab, an antibody targeting HER2, had an improved overall survival (OS). Based on the conclusive results of the ToGA trial, HER2 testing is considered a routine procedure for metastatic GC patients. Because only ~15% of GC cases are HER2-positive [7], there are several other targeted agents undergoing clinical trials. With respect to receptor tyrosine kinases (RTKs), phase III nimotuzumab trial (ENRICH) and rilotumumab trial (RILOMET-1) for epidermal growth factor receptor (EGFR)- and MET-positive GC are under investigation (ClinicalTrials.gov Identifier, NCT01813253 and NCT01697072). Ramucirumab, a monoclonal antibody against VEGFR2, improved OS when administered as monotherapy or in combination with paclitaxel as second-line chemotherapy [8, 9]. Apatinib, a small-molecule RTK inhibitor specific to VEGFR2, also improved OS when used as second-line or third-line treatment [10]. However, despite a rapid development of targeted therapies, no valid molecular targets and therapies have been identified with the exception of HER2. In addition, the expression of various molecular markers has not been simultaneously evaluated in a large GC cohort.

Recent studies have characterized GC as a heterogeneous disease, and defining molecular classification has become a main concern for GC research. Large-scale molecular profiling in GC as reported in The Cancer Genome Atlas (TCGA) and Asian Cancer Research Group (ACRG) provide excellent molecular classifications in addition to histopathology, which can be used as a guide for developing targeted agents [11, 12]. Despite classifications provided by TCGA and ACRG, it is still highly necessary to perform relevant assays that take into account molecular heterogeneity.

In the present study, we used clinically relevant immunohistochemistry (IHC) and in-situ hybridization (ISH) assays for comprehensive analyses of a large cohort of metastatic GC. We also evaluated the frequency and distributions of profiles to provide a practical guide for the targeted agents.

## RESULTS

### Sample set and classification

Of 438 Korean patients, 300 (68.5%) patients were male with a median age of 57 years (range 22-86). Majority of the tumors (65.1%) were located in the upper and middle stomach and 66.0% possessed undifferentiated histology. The histology of well differentiated (n = 29) and moderately differentiated (n=120) tubular adenocarcinoma were classified as differentiated, whereas poorly differentiated (n=223) and others (n=66) were classified as undifferentiated [13]. Approximately half of the 406 available cases were Bormann type III tumors. According to the recommended guideline [4, 14], adjuvant chemotherapy (xeloda/oxaliplatin or TS-1) was given in stage II/III patients. Among 217 stage II/III patients, 183 (84.3%) were treated with TS-1 or xeloda/oxaliplatin regimens.

**Figure 1:**
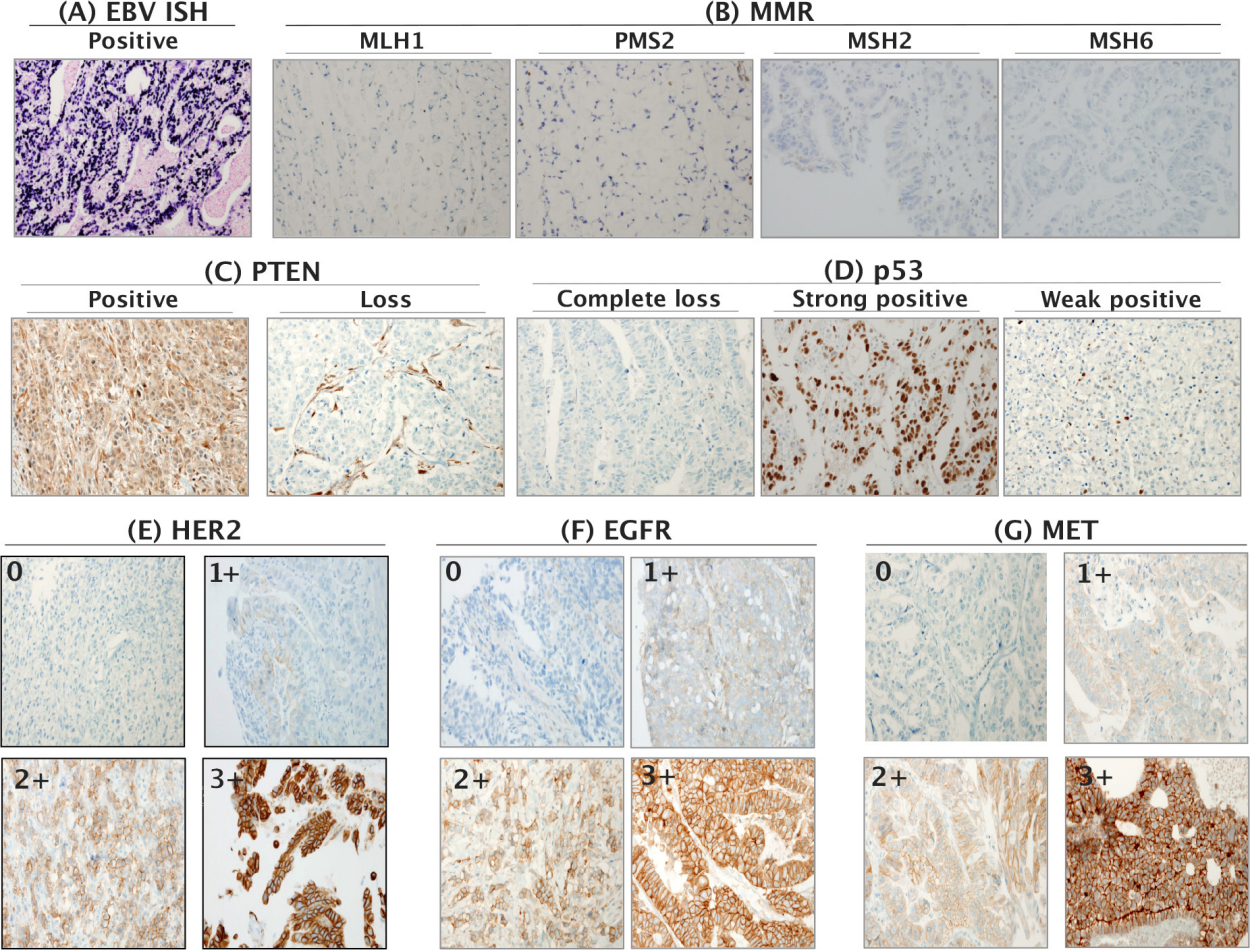
Representative positive images of multiple markers **A.** On EBER ISH, strong nuclear positivity is evident. **B.** MMR-deficient cancers have a loss of MLH1, PMS2, MSH2, and MSH6 expression. **C.** PTEN-positive and PTEN-loss staining. **D.** p53 is completely absent, strong positive and weakly positive. Typical 0, 1+, 2+, and 3+ expression of HER2 **E.** EGFR **F.** and MET **G.**

**Figure 2:**
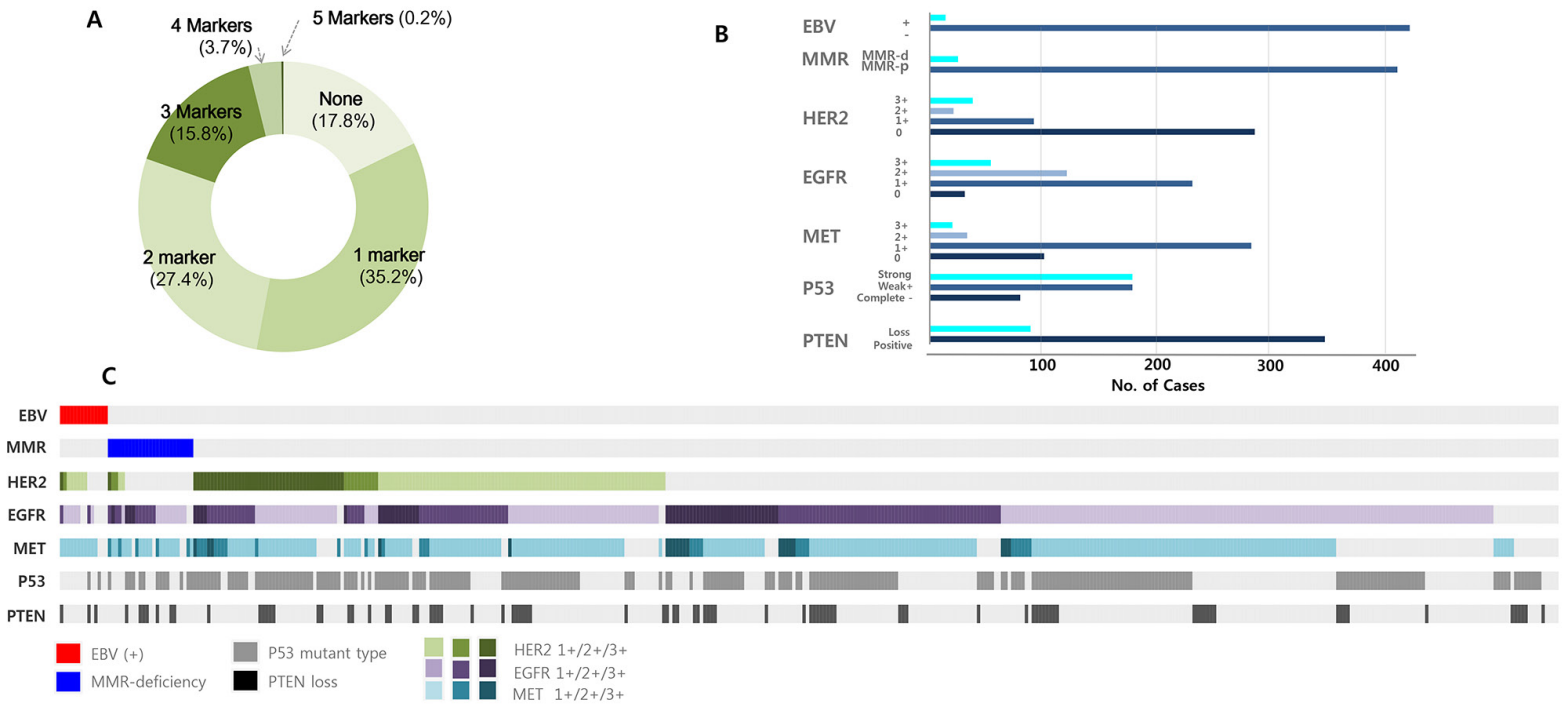
Patterns and frequencies of 10 molecular markers **A.** Profile of the number of positive markers. **B.** IHC/ISH expression of each marker is shown across samples. The vertical line shows the name and expression of the markers and the horizontal line indicates sample numbers. **C.** Integrated description of expression profiles.

We profiled 10 molecular markers using IHC and ISH (Figure 1). On average, we observed a median of 1 genomic aberration (range 0-5), comprising a mixture of each marker (Figure 2A). Of note, 1 tumor (0.2%) had 5 aberrations in PTEN, p53, EGFR, HER2, and MET. Epstein-Barr virus (EBV) was identified by ISH in 14 (3.3%) cases and MMR deficiency was demonstrated in 21 (4.8%). RTK overexpression occurred in 218 (49.8%) cases, in which EGFR was most frequently overexpressed (n=175, 39.9%), followed by HER2 (n=59, 13.5%) and MET (n=53, 12.1%) (Figure 2B). More than half of the observed cases had total loss of expression or strong positive expression for p53 staining, and 20.3% of cases exhibited loss of PTEN. MMR deficiency was mutually exclusive with EBV positivity. HER2 expression also showed a tendency towards exclusivity: the majority of HER2 3+ tumors (94.7%) were EBV-negative and MMR-proficient. There was only HER2 3+ tumor that was EBV positive, and another that was MMR deficient. A comprehensive description of each marker is illustrated in Figure 2C.

### Characteristics of EBV positive and MMR deficient GCs

IHC analysis revealed the presence of EBV in 14 (3.3%) cases, all of which also revealed the presence of EBER. The clinicopathologic features according to EBV and MMR status are summarized in Table 1. EBV-positive tumors were predominantly found in male patients (100% *vs* 67.4%, P=0.01), had an undifferentiated histology (92.9% *vs* 65.1%, P=0.03), and were proximal (85.7% vs 64.4%, P=0.04). There was no significant difference regarding metastatic site, Borrmann type, and stage.

**Table 1:** Clinicopathologic characteristics according to EBV and MMR expression

Characteristics	Total	EBV			MMR		
		Positive	Negative	*P* value	Deficient	Proficient	*P* value
**Total patients**	438	14 (3.3%)	424 (96.8%)		21 (4.8%)	417 (95.2%)	
**Age, years**				0.96			0.04
Median	57	57.5	57		63	56	
Range	22-86	43-75	22-86		31-80	22-86	
**Sex**							
Male	300 (68.5%)	14 (100%)	286 (67.4%)	0.01	14 (66.7%)	286 (68.6%)	0.85
Female	138 (31.5%)	0	138 (32.6%)		7 (33.3%)	131 (31.4%)	
**Metastatic site**							
Liver	73 (19.3%)	3 (4.1%)	70 (95.6%)	0.63	5 (6.8%)	68 (93.2%)	0.37
Lymph node	72 (19.0%)	1 (7.1%)	71 (94.9%)	0.34	2 (2.8%)	70 (97.2%)	0.38
Peritoneum	140 (36.9%)	5 (3.6%)	135 (96.4%)	0.76	3 (2.1%)	137 (97.9%)	0.04
Bone	21 (5.5%)	1 (4.8%)	20 (95.2%)	0.67	0	21 (100%)	0.29
Lung	13 (3.4%)	0	13 (100%)	0.51	0	13 (100%)	0.41
Brain	2 (0.5%)	0	2 (100%)	0.79	0	2 (100%)	0.75
Others	58 (15.3%)	2 (14.3%)	56 (85.7%)	0.91	2 (3.4%)	56 (96.6%)	0.61
**Location**							
Upper/middle	285 (65.1%)	12 (85.7%)	273 (64.4%)	0.04	1 (4.8%)	49 (11.8%)	0.33
Lower	153 (34.9%)	2 (14.3%)	151 (35.6%)		20 (95.2%)	368 (88.2%)	
**Histology**							
Differentiated	149 (34.0%)	1 (7.1%)	148 (34.9%)	0.03	12 (57.1%)	137 (32.9%)	0.02
Undifferentiated	289 (66.0%)	13 (92.9%)	276 (65.1%)		9 (42.9%)	280 (67.1%)	
**Lauren classification (n=245)**							
Intestinal	98 (40.0%)	3 (33.3%)	95 (40.3%)	0.84	9 (60.0%)	89 (38.7%)	0.25
Diffuse	130 (53.1%)	5 (55.6%)	125 (52.9%)		5 (33.3%)	125 (54.3%)	
Mixed	17 (6.9%)	1 (11.1%)	16 (6.8%)		1 (6.7%)	16 (7.0%)	
**Borrmann (n=406)**							
1	17 (4.2%)	1 (7.1%)	16 (4.1%)	0.59	1 (5.0%)	16 (4.1%)	0.05
2	71 (17.5%)	4 (28.6%)	67 (17.1%)		5 (25.0%)	66 (17.1%)	
3	223 (54.9%)	7 (50.0%)	216 (55.1%)		14 (70.0%)	209 (54.1%)	
4	95 (23.4%)	2 (14.3%)	93 (23.7%)		0	95 (24.6%)	
**Stage**							
I	23 (5.3%)	0	23 (5.4%)	0.59	1 (4.3%)	22 (5.3%)	0.44
II	87 (19.9%)	3 (21.4%)	84 (19.8%)		7 (33.3%)	80 (19.2%)	
III	130 (29.7%)	6 (42.9%)	124 (29.2%)		6 (28.6%)	124 (29.7%)	
IV	198 (45.2%)	5 (35.7%)	193 (45.5%)		7 (33.3%)	191 (45.8%)	
**MSI**							
MMR-proficient	417 (95.2%)	14 (100%)	403 (95.0%)	0.39	−	−	−
MMR-deficient	21 (4.8%)	0	21 (5.0%)		−	−	

MMR deficiency was defined as loss of expression in one of MLH1, PMS2, or MSH6. Among 21 cases of MMR deficiency, 15 (71.4%) had simultaneous loss of MLH1 and PMS2 expression, two (9.5%) had a simultaneous loss of MSH2 and MSH6, and one (4.7%) had loss of both MSH6 and PMS2. Two cases (9.5%) and one case (4.7%) had a loss of only MSH6 and PMS2, respectively. The MMR-deficient group consisted of older patients (63 *vs* 56 years, P=0.08), the tumors had a differentiated histology (57.1% v*s* 32.9%, P=0.02), and fewer instances of peritoneal seeding than the MMR-proficient group (14.3% vs 32.9%, P=0.04).

### Characteristics of RTK overexpression

**Figure 3:**
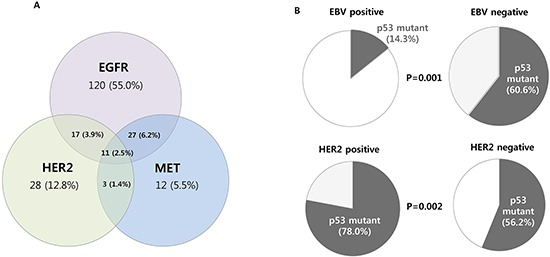
Correlative description for different markers. **A.** Diagram showing the number of dysregulated RTKs **B.** The proportion of p53- tumors harboring EBV and HER2 -positivity.

Scores of 0 and 1+ were regarded as negative and scores of 2+ and 3+ as positive and no positivity for any of the 3 RTKs were seen in 220 (50.2%) samples. Among 218 (49.8%) cases with any positive RTKs, 11 cases (2.5%) had simultaneous positive expression of HER2, EGFR, and MET (Figure 3A): three cases were scored as 3+/3+/3+ and eight cases scored 3+/3+/2+ for EGFR/HER2/MET expression. Forty-seven (10.7%) tumors had simultaneous positive expression of two RTKs: 27 (6.2%) with EGFR/MET, 17 (3.9%) with EGFR/HER2, and 3 (1.4%) with HER2/MET.

Table 2 summarizes RTK protein expression and the clinicopathological findings. With respect to HER2 expression, 287 (65.6%), 92 (21.0%), 21 (4.8%), and 38 (8.7%) cases were scored as 0, 1+, 2+, and 3+, respectively. Among those with a score of 2+, silver ISH analysis revealed HER2 amplification in eight cases and they were finally described as HER2 positive cases. HER2-positive tumors were identified in older patients (60 *vs* 56 years, P=0.04); had a higher instance of liver metastasis (30.5% *vs* 14.5%, P<0.001), lymph node metastasis (25.4% *vs* 15.0%, P=0.04), and lung metastasis (8.5% *vs* 2.1%, P=0.01); and were found in a lower location (49.2% vs 32.7%, P=0.01). HER2 positivity was higher in patients with intestinal type GC (62.1% *vs* 37.0%, P=0.03) and a differentiated histology (59.3% *vs* 30.1%, P<0.001). However, there was no significant correlation between HER2 overexpression with Borrman type and stage.

**Table 2:** Clinicopathological characteristics of each RTKs overexpression

Characteristics		HER2					EGFR					MET				
		Positive*		Negative		*P*	Positive		Negative		*P*	Positive		Negative		*P*
		3+	2+	1+	0		3+	2+	1+	0		3+	2+	1+	0	
**Total (%)**	438	46 (10.5)	13 (3.0)	92 (21.0)	287 (65.5)	−	54 (12.3)	121 (27.6)	232 (53.0)	31 (7.1)		20 (4.6)	33 (7.5)	284 (64.8)	101 (23.1)	−
**Age, years**																
Median	57	60		56		0.04	58		56		0.21	58		56		0.26
Range	22-86	22-82		23-86			22-86		23-85			29-86		22-86		
**Sex**																
Male	300	44 (50.8%)		256 (67.5%)		0.28	129 (73.7%)		171 (65.0%)		0.04	31 (58.5%)		269 (69.9%)		0.1
Female	138	15 (49.2%)		123 (32.5%)			46 (26.3%)		92 (35.0%)			22 (41.5%)		116 (30.1%)		
**Metastatic site**																
Liver	73	18 (24.7%)		55 (75.3%)		<0.001	30 (41.1%)		43 (58.9%)		0.83	10 (13.7%)		63 (86.3%)		0.65
Lymph node	72	15 (20.8%)		57 (79.2%)		0.04	35 (48.6%)		37 (51.4%)		0.10	16 (22.2%)		56 (77.8%)		<0.001
Peritoneum	140	13 (9.3%)		127 (33.5%)		0.08	51 (36.4%)		89 (63.6%)		0.30	13 (9.3%)		127 (90.7%)		0.22
Bone	21	3 (14.3%)		18 (85.7%)		0.91	8 (38.1%)		13 (61.9%)		0.86	3 (14.3%)		18 (85.7%)		0.75
Lung	13	5 (38.5%)		8 (61.5%)		0.01	9 (69.2%)		4 (30.8%)		0.03	2 (15.4%)		11 (84.6%)		0.71
Brain	2	0		2 (100%)		0.57	2 (100%)		0		0.08	0		2 (100%)		0.59
Others	58	9 (15.5%)		49 (84.5%)		0.62	21 (36.2%)		37 (63.8%)		0.53	4 (6.9%)		54 (93.1%)		0.19
**Location**																
Upper/middle	285	30 (50.8%)		255 (67.3%)		0.01	106 (60.6%)		179 (68.1%)		0.10	32 (60.4%)		253 (65.7%)		0.45
Lower	153	29 (49.2%)		124 (32.7%)			69 (39.4%)		84 (31.9%)			21 (39.6%)		132 (34.3%)		
**Histology**^†^																
Diff	149	35 (59.3%)		114 (30.1%)		<0.001	71 (40.6%)		78 (29.7%)		0.02	19 (35.8%)		130 (33.8%)		0.76
Undiff	289	24 (40.7%)		265 (69.9%)			104 (59.4%)		185 (70.3%)			34 (64.2%)		255 (66.2%)		
**Lauren classification (n=245)**																
Intestinal	98	18 (62.1%)		80 (37.0%)		0.03	51 (53.1%)		47 (31.5%)		<0.001	10 (47.6%)		88 (39.3%)		0.73
Diffuse	130	10 (34.5%)		120 (55.6%)			39 (40.6%)		91 (61.1%)			10 (47.6%)		120 (53.6%)		
Mixed	17	1 (3.4%)		16 (7.4%)			6 (6.3%)		11 (7.4%)			1 (4.8%)		16 (7.1%)		
**Borrmann (n=406)**																
1	17	2 (3.5%)		15 (4.3%)		0.84	6 (3.6%)		11 (4.6%)		0.1	2 (4.3%)		15 (4.2%)		0.28
2	71	10 (17.5%)		61 (17.5%)			31 (18.6%)		40 (16.7%)			12 (25.5%)		59 (16.4%)		
3	223	34 (59.6%)		189 (54.2%)			101 (60.5%)		122 (51.0%)			20 (42.6%)		203 (56.5%)		
4	95	11 (19.3%)		84 (24.1%)			29 (17.4%)		66 (27.6%)			13 (27.7%)		82 (22.8%)		
**Stage**																
I	23	0		23 (6.1%)		0.02	10 (5.7%)		13 (4.9%)		0.95	4 (7.5%)		19 (4.9%)		0.02
II	87	6 (10.2%)		81 (21.4%)			34 (19.4%)		53 (20.2%)			4 (7.5%)		83 (21.6%)		
III	130	18 (30.5%)		112 (29.6%)			50 (28.6%)		80 (30.4%)			12 (22.6%)		118 (30.6%)		
IV	198	35 (59.3%)		163 (43.0%)			81 (46.3%)		117 (44.5%)			33 (62.3%)		165 (42.9%)		

* All IHC score of 3+ or a score of 2+ plus HER2 gene amplification was defined as HER2 3+.

† Histology: diff; differentiated, undiff; undifferentiated

A total of 31 (7.1%), 232 (53.0%), 121 (27.6%), and 54 (12.3%) cases were scored as 0,1+, 2+, and 3+ with respect to EGFR expression. EGFR-positive cases arose predominantly identified in male patients (73.7% vs 65.0%, P=0.04), and in conjunction with instances of lung (P=0.03) and brain metastasis (P=0.08). EGFR positivity was also associated a differentiated histology (40.6% vs 29.7%, P=0.02) and intestinal type GC (53.1% vs 31.5%, < P=0.001).

Finally, with respect to MET expression, 101 (23.1%) cases earned a score of 0, 284 (64.8%) scored 1+, 33 (7.5%) scored 2+, and 20 (4.6%) scored 3+. The MET-positive group was more frequently associated with lymph node metastasis than the MET-negative group (30.2% vs 14.5%, P<0.001). However, there was no significant correlation between MET overexpression and location, histology, or Lauren classification.

### PTEN and p53 altered GCs

With respect to p53 expression, 80 cases (18.3%) had a complete loss of expression and 179 (40.9%) had diffuse strong expression. By combining those two immunohistochemical staining patterns (complete loss and strong-diffuse expression) [15], 259 cases (59.1%) were identified as overexpression/null tumors (Table 3). The other 179 (40.9%) cases had characteristics of focal and weak staining. The overexpression/null group appeared among older (57 *vs* 55 years, P=0.03) and predominantly male patients (73.7% *vs* 60.9%, P<0.01), and had a differentiated histology (39.0% *vs* 26.8%, P=0.01). Furthermore, p53 overexpression/null tumors was associated with the EBV-negative (60.6% *vs* 14.3%, P=0.001) and HER2 positive tumors (78.0% vs 56.2%, P=0.002, Figure 3B).

**Table 3:** Clinicopathological characteristics according to p53 and PTEN expression

Characteristics	Total	p53			PTEN		
		Overexpression/null	Weak	*P* value	Loss	Positive	*P* value
**Total (%)**	438	259 (59.1%)	179 (40.9%)		89 (20.3%)	349 (79.7%)	
**Age, years**							
Median	57	57	55	0.03	57	56	0.53
Range	22-86	22-86	29-82		27-83	22-86	
**Sex**							
Male	300	191 (73.7%)	109 (60.9%)	<0.01	69 (77.5%)	231 (66.2%)	0.04
Female	138	68 (26.3%)	70 (39.1%)		20 (22.5%)	118 (33.8%)	
**Metastatic site**							
Liver	73	48 (65.8%)	25 (34.2%)	0.21	18 (24.7%)	55 (75.3%)	0.31
Lymph node	72	50 (69.4%)	22 (30.6%)	0.05	18 (25.0%)	54 (75.0%)	0.28
Peritoneum	140	75 (53.6%)	65 (46.4%)	0.11	27 (19.3%)	113 (80.7%)	0.71
Bone	21	12 (57.1%)	9 (42.9%)	0.85	2 (9.5%)	19 (90.5%)	0.21
Lung	13	9 (69.2%)	4 (30.8%)	0.45	4 (30.8%)	9 (69.2%)	0.34
Brain	2	1 (50%)	1 (50%)	0.79	1 (50%)	1 (50%)	0.29
Others	58	33 (56.9%)	25 (43.1%)	0.71	11 (19.0%)	47 (81.0%)	0.78
**Location**							
Upper/middle	285	167 (64.5%)	118 (65.9%)	0.75	55 (61.8%)	230 (65.9%)	0.47
Lower	153	92 (35.5%)	61 (34.1%)		34 (38.2%)	119 (34.1%)	
**Histology**							
Differentiated	149	101 (39.0%)	48 (26.8%)	0.01	37 (41.6%)	112 (32.1%)	0.09
Undifferentiated	289	158 (61.0%)	131 (73.2%)		52 (58.4%)	237 (67.9%)	
**Lauren classification (n=245)**							
Intestinal	98	66 (45.8%)	32 (31.7%)	0.07	21 (53.8%)	77 (37.4%)	0.02
Diffuse	130	70 (48.6%)	60 (59.4%)		16 (41.0%)	114 (55.3%)	
Mixed	17	8 (5.6%)	9 (8.9%)		2 (5.1%)	15 (7.3%)	
**Borrmann (n=406)**							
1	17	8 (3.3%)	9 (5.4%)	0.62	5 (6.0%)	12 (3.7%)	0.56
2	71	42 (17.6%)	29 (17.4%)		11 (13.1%)	60 (18.6%)	
3	223	136 (56.9%)	87 (52.1%)		48 (57.1%)	175 (54.3%)	
4	95	53 (22.2%)	42 (25.1%)		20 (23.8%)	75 (23.3%)	
**Stage**							
I	23	17 (6.6%)	6 (3.4%)	0.05	8 (9.0%)	15 (4.3%)	0.07
II	87	50 (19.3%)	37 (20.7%)		12 (13.5%)	75 (21.5%)	
III	130	76 (29.3%)	54 (30.2%)		23 (25.8%)	107 (30.7%)	
IV	198	116 (44.8%)	82 (45.8%)		46 (51.7%)	152 (43.6%)	
**PTEN**							
Loss	89	63 (24.3%)	26 (14.5%)	<0.01			
Positive	349	196 (75.7%)	153 (85.5%)				

We observed PTEN loss in 89 patients (20.3%) and PTEN-positive expression in 349 patients (79.7%). PTEN loss was predominant in male patients (77.5% vs 66.2%, P=0.04). However, PTEN status had no significant association with age, metastatic site, location, and histology. Finally, PTEN loss was significantly associated with p53 overexpression/null group (24.3% *vs* 14.5%, P<0.01).

## DISCUSSION

In this study, we described feasible and comprehensive molecular profiles that define specific subtypes of GC using IHC and ISH assays and that have practical applications in a clinical setting. These molecular platforms will provide a convenient screening tool to classify GC. It may facilitate the development and enrollment of clinical trials with targeted agents, ultimately improving the survival outcome for patients with GC.

Previously, TCGA categorized GC into four molecular genomic subtypes: EBV-positive tumors (8.8%), microsatellite instability-high (MSI-H) tumors (21.6%), genomically stable tumors (GS, 19.6%), and tumors with chromosomal instability (CIN, 49.6%) [11]. In addition, they identified key druggable targets for each subgroup: *PIK3CA, JAK2,* and *PD-L1/PD-L2* for EBV-positive tumors; *PIK3CA* and *ERBB2/3* for MSI; *RHOA* and *CDH1* for GS, and RTKs for CIN. This classification of distinct molecular features provided valuable guidelines for developing therapeutic strategies. Recently, the ACRG used primary gastrectomy samples to characterize 4 subtypes of GC linked to distinct patterns of molecular alterations and prognosis: MSI-H tumors (22.7%), which have the best prognosis; microsatellite stable (MSS)/epithelial to mesenchymal transition (EMT) tumors (15.3%), characterized by loss of *CDH1* and worst prognosis; and MSS/TP53 active (26.3%) and inactive (35.7%) tumors, both of which have an intermediate prognosis [12]. They also validated these subtypes in independent cohorts and showed that they are associated with distinct patterns of genomic alterations in addition to prognosis.

Although there have been important findings from recent molecular classifications that have enhanced our understanding of GC biology, more work is needed to establish the clinical relevance. Firstly, there is a great amount of diversity among sample population. In the TCGA cohort, only 47.7% (295 out of 618 cases) of initial samples were analyzed and included in the data set due to quality issues and majority of these patients (92.5%) had non-metastatic cancer. Because personalized treatment is geared towards targeting metastatic or recurrent GC, it is challenging to adapt the data acquired from the selected and resected cases for clinical relevance. In addition, as compared with the Asian cohort in ACRG, only 19.7% of TCGA cases are from Asian populations (12.9% in Korean and 6.8% in Vietnam). Therefore, because distributions of race and stages are known to have significant impact on the outcome of molecular profiling [16], any conclusions derived from these studies may not be universally relevant. Secondly, TCGA and ACRG data were analyzed from different profiling platforms with fresh and large tumor samples, so it is difficult to apply their observations in metastatic GC with FFPE samples. Therefore, reliability of molecular aberrations remains questionable unless their performance is validated in a clinical setting by an applicable method, such as HER2 testing.

In our study, we developed a GC panel incorporating TCGA and ACRG classification: EBV ISH for EBV-positivity, MMR protein (MLH1, PMS2, MSH2, and MSH6) for MSI tumors, druggable RTKs (HER2, EGFR, MET), PI3K pathway component (PTEN), and p53 protein. Our IHC/ISH results are consistent with previous comprehensive molecular studies. Like EBV positive cases were enriched in MSS/TP53^+^ (intact TP53) in ACRG subtypes, all EBV positive cases in our study also showed MMR proficient and mostly p53 wild pattern. Therefore, we classified GCs using the TCGA/ACRG genomic scheme, and were able to identify a great amount of consistency with these background studies.

We observed 3.3% of EBV-positivity and 4.8% of MMR-deficiency. With respect to EBV-positivity, our observations of predominantly male patients, undifferentiated histology, and upper anatomical location are in agreement with those of previous studies [17–19]. Our observation of an inverse correlation between EBV and p53 is also consistent with previous studies [20, 21]; an increased frequency of mutant-pattern p53 expression was found in EBV-negative carcinomas. In addition, based on high concordance rate with PCR-based assays [22–24], we identified distinct clinicopathologic features (older patients and differentiated histology) of MMR-deficient GCs [24–26]. TCGA reported higher frequency of EBV-positivity (8.8%) and MSI-H (21.6%) compared to our findings. In this study, we found 3.3% of tumors were EBV-positive, which is similar to the data reported in Korean (5.6%) and Japanese (6.4%) cohorts [27, 28]. Given that EBV-positivity and MMR-deficiency is more common in early stages of GC [29, 30], the inclusion of advanced stages in the study may have caused a slightly lower frequency. Recently, treatment with pembrolizumab, an anti–programmed death 1 (PD-1) immune checkpoint inhibitor, was found to have a dramatic and durable response for MMR-deficient tumors but not for MMR-proficient tumors (62% vs 0%) [31]. In addition, TCGA data also showed high PD-L1/L2 expression in an EBV-positive subgroup. Therefore, both EBV-positive and MMR-deficient GCs (up to 10% of metastatic GC) need strong attention for the immune modulating agents with sensitive and cost-effective screening assay.

Although several RTKs have been widely investigated in GC, only a few studies evaluated simultaneous profiles of RTKs in a large number of GC. Lennerz *et al.* reported mutually exclusive expression of HER2, MET, and EGFR using FISH assay [32]. Dent *et al.* also showed that EGFR, HER2, MET, and FGFR2 were amplified in GC using an SNP [33]. Recently, protein expression (using IHC) and copy numbers (using next-generation sequencing) have been used, and high-level amplification was correlated with protein expression in mutually exclusive way [34, 35]. In our study, although several RTKs were overexpressed simultaneously, 73.4% (160 out of 218) of cases had overexpression of only one RTK. Therefore, IHC can provide meaningful information that can be applied towards screening in a clinical setting.

From a therapeutic perspective, high RTK amplification levels may serve as reliable targets. Although lapatinib treatment did not significantly improve survival for *HER2*-amplified GC patients (TyTAN study), both *HER2* amplified (by FISH) and IHC 3+ cases had significantly better progression free survival (HR 0.59, P=0.0176) and overall survival (HR 0.54, P=0.01) [36]. In patient-derived xenograft models of GC, cases with *EGFR* amplification and overexpression (3+) benefitted from cetuximab treatment, an EGFR-directed monoclonal antibody [37]. High levels of RTK amplification show high protein overexpression using IHC in most cases of GC [35]. Because amplifications occur at a low frequency, it is important to develop a reliable IHC assay to screen the right population for RTK inhibitors. With the exception of HER2, discrepancies between various studies may be attributed to the lack of standardized IHC protocols and evaluations. Therefore, these criteria need further validation in order to contribute towards future clinical trials with targeted agents.

Loss of PTEN, a tumor suppressor gene, can be caused by mutations, gene deletion, and promoter hypermethylation. The robustness of IHC assays in the detection of PTEN loss has been studied widely in various tumors [38–40]. In the event of haploinsufficiency accompanied by second allele inactivation, 30% of PTEN protein loss cases are not detectable by FISH analysis [41, 42]. Therefore, IHC is a robust and reliable assay for PTEN loss. As for its therapeutic relevance, PTEN loss is known to contribute towards poor response to HER2-targeted agents in breast and gastric cancer [43–45]. To address this, there are clinical trials underway that combine a PI3K inhibitor with trastuzumab (clinicaltrial.gov; NCT01589861 and NCT01471847). The clinical benefit of GSK2636771, a selective PI3Kβ inhibitor, was also studied for PTEN-deficient solid tumors [46], and trials combining it with paclitaxel are underway for PTEN-deficient gastric cancers using IHC assay (clinicaltrial.gov identifier; NCT02615730).

Our study may have important clinical implications for GC. First, our classification incorporates the two genomic schemes set by TCGA and ACRG. Our GC panel provides a valuable foundation to refine molecular classification and tailored therapies in advanced GC. Second, two major obstacles of addressing molecular signatures in practice are cost effectiveness and tumor status; TCGA used six molecular analyses platform including whole-exome sequencing, RNA sequencing, and protein arrays. In our study, we used 10 slides with IHC/ISH assays, which can be easily used with archival biopsy tissue.

In conclusion, we carried out a comprehensive analysis using IHC/ISH to classify optimal subgroups of GC. Our efficient screening assay will facilitate the development of successful future clinical trials using molecular targeted agents.

## MATERIALS AND METHODS

### Patients and tissue samples

This study was conducted with a consecutive cohort of 438 patients with advanced gastric adenocarcinoma who underwent palliative chemotherapy at Severance Hospital, Seoul, Korea between January 2014 and October 2015. A total of 438 formalin-fixed, paraffin-embedded (FFPE) tissue blocks from primary stomach were available for examination along 10 IHC profiles. All diagnoses were reviewed by two experienced pathologist (H.K. and S.J.S) and confirmed by hematoxylin and eosin staining.

Patients’ information was collected by reviewing the medical records for evaluation of clinicopathologic characteristics and survival outcome. Staging was determined using the 7th edition American Joint Committee on Cancer guideline of tumor, node, and metastasis (TNM) classification. The study was approved by the institutional review board of Severance Hospital.

### Immunohistochemical staining

IHC was performed with a Ventana XT automated staining instrument. Antibodies recognizing the following targets were used: MutL homolog 1 (MLH1, ready to use, clone M1, Roche, Basel, Schweiz), MutS protein homolog 2 (MSH2, ready to use, clone G219-1129, Roche), MutS homolog 6 (MSH6, 1:100, clone 44, Cell Marque, Rocklin, CA), postmeiotic segregation increased 2 (PMS2, 1:40, clone MRQ28, Cell Marque), HER2 (ready to use, clone 4B5, Roche), EGFR (1:100, EP38Y, Abcam, Cambridge, UK), c-MET (ready to use, clone SP44, Roche), PTEN (1:100, clone 138G6, Cell signaling, Danvers, MA), and p53 (1:300, DO7, Novocastra, Newcastle, UK). Sections were deparaffinized using EZ Prep solution (Ventana Corporation, Tucson, AZ). CC1 standard (pH 8.4 buffer containing Tris/borate/EDTA) was used for antigen retrieval and blocked with inhibitor D (3% H_2_O_2_) for 4 min at 37°C. Slides were incubated with primary antibody for 40 min at 37°C, followed by a universal secondary antibody for 20 min at 37°C. Slides were incubated in streptavidin-horseradish peroxidase (SA-HRP) D for 16 min at 37°C, after which the substrate, 3,3′-diaminobenzidine tetrahydrochloride (DAB) H_2_O_2_, was added for 8 min, followed by hematoxylin and bluing reagent counterstaining at 37°C.

### EBER-in situ hybridization (ISH)

EBER (Epstein-Barr virus-encoded small RNAs) ISH was performed using a Ventana Benchmark ISH system (Ventana ISH iView kit, Ventana Corporation, Tucson, AZ, USA). Paraffin-embedded tissue sections were deparaffinized using EZ Prep buffer (Ventana Corporation), and then digested with Protease I for 4 min. Probes were added to the sample, and then denaturation was performed at 85°C (10 min), followed by hybridization at 37°C (1 hour). The probes labeled with fluorescein contained a cocktail of oligonucleotides dissolved in a formamide-based diluent. After hybridization, tissues were washed 3 times using 2x saline sodium citrate buffer at 57°C. Incubation with anti-fluorescein monoclonal antibody was performed for 20 min and then the alkaline blue detection kit (Ventana Corporation) was used according to the manufacturer's protocol. The slides were counterstained with nuclear fast red (Ventana Corporation) for 10 min.

### Evaluation

HER2 staining was analyzed according to the system using the following parameters [47] : 0 was defined as no reactivity, or membranous reactivity, in <5 of tumor cells (for biopsy specimens) or <10% of tumor cells (for surgical specimens), 1+ staining was defined as faint/barely perceptible membranous reactivity in ≥5 cells or ≥10% of tumor cells; 2+ staining was defined as weak to moderate total or basolateral membranous reactivity in ≥5 cells or ≥10% of tumor cells; and 3+ staining as defined as moderate to strong total or basolateral membranous reactivity in ≥5 cells or ≥10% of tumor cells. For assessing expression levels of MET and EGFR, we compared them with the expression levels found in non-neoplastic epithelial cells. These samples were scored as either 0 (negative), 1+ (weaker or same as non-neoplastic epithelial cells), 2+ (moderately stronger than non-neoplastic epithelial cells), or 3+ (much stronger than non-neoplastic epithelial cells). Samples with an IHC score of 3+, and a score of 2+ plus HER2 gene amplification, were defined as having HER2 overexpression. For EGFR and MET, we defined an IHC score of 2+ and 3+ as overexpression based on previous studies [48, 49].

A loss of mismatch repair (MMR) protein expression was designated when none of the neoplastic cells showed nuclear staining, whereas normal expression was defined as the presence of nuclear expression of tumor cells, irrespective of the proportion or intensity. For evaluation of the PTEN protein, histology (H) scores were applied as follows: nuclear or cytoplasmic staining was scored as either 0 (negative), 1+ (weak), 2+ (moderate), or 3+ (strong). The percentage of cells at different staining intensities was determined by visual assessment, with the score calculated using the formula 1 × (% of 1+ cells) + 2 × (% of 2+ cells) + 3 × (% of 3+ cells). Samples were then classified as either loss (H-score < 100) or intact (≥100) for PTEN protein expression. For the p53 protein, the nuclear staining was taken into account, and it was scored as follows: wild pattern, in which tumor showed patch (≤ 50% of tumor cells); weak positive staining; and the tumor showed diffuse strong nuclear positive staining ( > 50% of tumor cells), or complete loss of expression.

### Statistical analysis

The correlation between marker status and clinical significance was assessed using the Χ^2^ test. All the tests were two-sided, and *P* values of <0.05 were considered significant. All statistical analysis was performed using SPSS version 18.0 (IBM, Chicago, IL).
